# 0889. Mitochondrial dysfunction contributes to sepsis-induces acute kidney injury

**DOI:** 10.1186/2197-425X-2-S1-O18

**Published:** 2014-09-26

**Authors:** E Greco, N Arulkumaran, M Sixma, H Courtneidge, M Duchen, F Tam, RJ Unwin, M Singer

**Affiliations:** Bloomsbury Institute of Intensive Care Medicine, University College London, London, UK; Department of Nephrology, Imperial College, London, UK; Centre for Nephrology, University College London, London, UK; Department of Cell and Developmental Biology, University College London, London, UK

## Introduction

Mechanisms underlying sepsis-induced acute kidney injury remain uncertain. Mitochondrial dysfunction is a hallmark of established sepsis, and may contribute to renal dysfunction.

## Objectives

To determine the effect of septic serum on renal tubular epithelial cell mitochondrial physiology.

## Methods

Multiphoton confocal microscopy images were recorded over 45 minutes in intact renal sections (taken from healthy rats) incubated in either physiologic saline solution (PSS) (n=6), sham serum (n=3), septic serum (n=3), and septic serum co-incubated with 10nM of the anti-oxidant 4-OH-Tempo (n=4). Pooled septic serum was obtained from (n=5) rats 24 hrs after induction of faecal peritonitis. Sham serum was obtained from 3 healthy controls. The percentage change in NADH redox state (using NADH autofluorescence), reactive oxygen species (ROS) production (using tetramethyl rhodamine methyl ester, TMRM) and mitochondrial membrane potential (using dihydroethidium) are expressed as ratios compared to baseline values. Wilcoxon Rank Sum test was used to compare groups with p< 0.05 taken as significant.

## Results

**Figure 1:**
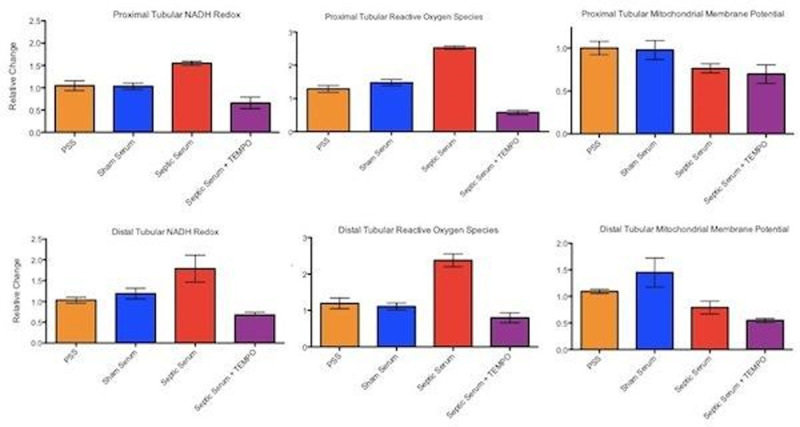
Figure 1

Mitochondrial dysfunction was seen in both proximal and distal tubular epithelial cells incubated in septic serum. NADH became more reduced, reactive oxygen species increased and mitochondrial membrane potential fell compared to either sham serum or PSS. The increases in NADH reduction and ROS production (but not mitochondrial membrane potential) on exposure to septic serum could be prevented by co-incubation with 4-OH-Tempo (Figure 1).

## Conclusions

Circulating mediators within septic serum can induce mitochondrial dysfunction in healthy renal tubular cells. Co-incubation with an anti-oxidant offered protection, suggesting these mediators are likely to be oxygen or nitrogen reactive species.

